# Physical and Psychological Effects Related to Food Habits and Lifestyle Changes Derived from COVID-19 Home Confinement in the Spanish Population

**DOI:** 10.3390/nu12113445

**Published:** 2020-11-10

**Authors:** Miguel López-Moreno, Maria Teresa Iglesias López, Marta Miguel, Marta Garcés-Rimón

**Affiliations:** 1 Instituto de Investigación en Ciencias de Alimentación (CIAL; CSIC-UAM), 28049 Madrid, Spain; miguel.lopez@csic.es (M.L.-M.); marta.miguel@csic.es (M.M.); 2 Grupo de Investigación en Biotecnología Alimentaria, Universidad Francisco de Vitoria, Pozuelo de Alarcón, 28223 Madrid, Spain; m.iglesias.prof@ufv.es

**Keywords:** COVID-19, food intake, lifestyle, emotional eating, home confinement, lockdown

## Abstract

As a consequence of COVID-19, millions of households have suffered mobility restrictions and changes in their lifestyle over several months. The aim of this study is to evaluate the effects of COVID-19 home confinement on the food habits, lifestyle and emotional balance of the Spanish population. This cross-sectional study used data collected via an anonymous online questionnaire during the month before lockdown finished in Spain, with a total of 675 participants. 38.8% of the respondents experienced weight gain while 31.1% lost weight during confinement. The increase in body weight was positively correlated with age (Rs = 0.14, *p* < 0.05) and BMI (Rs = 0.20, *p* < 0.05). We also identified that 39.7% reported poorer quality sleep, positively correlated with BMI (Rs = −0.18, *p* < 0.05) and with age (Rs = −0.21, *p* < 0.05). 44.7% of the participants had not performed physical exercise during confinement with differences by sex (*p* < 0.05), by age (*p* < 0.05), by BMI (*p* < 0.05) and by sleep quality (*p* < 0.05). According to an emotional-eater questionnaire, 21.8% and 11% were classified as an emotional eater or a very emotional eater, respectively. We emphasize the importance of adopting a healthy lifestyle, as the COVID-19 pandemic is ongoing.

## 1. Introduction

Infectious diseases are growing in this century and one of its greatest challenges is the continuing global impact of these illnesses [1]. After Severe Acute Respiratory Syndrome (SARS) in 2002 and Ebola and Middle East Respiratory Syndrome (MERS) in 2015, the beginning of 2020 was marked by a novel coronavirus global outbreak [2,3]. The novel coronavirus disease, named COVID-19 by the World Health Organization (WHO), has caused severe acute respiratory syndrome coronavirus 2 (WHO, 2020). This pathogen was later renamed SARS-CoV-2 by the Coronavirus Study Group [4].

COVID-19 was first reported in Wuhan city (Hubei, China) in early December 2019, and rapidly spread worldwide. In January 2020, the WHO declared the global outbreak as a Public Health Emergency of International Concern [5], and in March 2020 it was declared as a global pandemic [6]. According to data from the Johns Hopkins University, by 22 July COVID-19 had seen 15,016,440 cases and 618,001 deaths worldwide. Spain is in the seventh worst position on a global scale, with 896,086 cases, and 33,204 deaths (John Hopkins University, 2020 (accessed on 14 October 2020)).

This high infectivity and spread have been worrying the health authorities and the general population. Because of the absence of specific vaccines or treatments, government authorities, following WHO recommendations, were forced to declare a health alarm status and order the confinement of citizens. People in most countries were put under quarantine, with more or less rigor, in order to reduce the spread of the virus, which then lessens the impact on medical resources [7]. In Spain, around 47,329,981 people suffered mobility restrictions and changes in their lifestyle during the state of alarm from 14 March 2020 to 21 June 2020 [8].

Quarantine is associated with the interruption of free movement and work routine, and could result in mood changes, such as anxiety or boredom. These emotional changes are associated with a greater energy intake, as well as the consumption of higher quantities of macronutrients [9,10], linked not only with confinement, but also with the economic decline, the uncertain situation and the increase in physical inactivity [11].

Eating is a response that takes place due to nutritive and non-nutritive signs within the organism. Increased time at home may provoke additional eating, especially in people with obesity, who often show an oversensitivity to non-nutritive signs (social, emotional, or conditioned craving for certain foods), and a concomitant desensitization, mainly related to normal satiety processes [12,13]. Furthermore, the constant bombardment of news and information in the media about the COVID-19 pandemic can be stressful. Consequently, this stress pushes people toward overeating, mainly by looking for food that helps to release stress, the so-called “comfort foods” [14], mainly composed of caloric nutrients such as sugars or fats. This desire to consume a specific kind of food is defined as “food craving” [15]. The satisfaction of carbohydrate craving is related to serotonin production and, in consequence, a positive effect on mood. However, abuse of carbohydrate or fat intake in the diet could increase the risk of developing obesity that, as well as being a chronic state of inflammation, has been demonstrated to increase the risk of serious complications with COVID-19 [16]. Stress related to the confinement also results in sleep disturbances which could increase food intake [17]. Furthermore, during confinement physical activity is often reduced, which can result in the increase of stress. 

In view of the current situation, and thinking of future re-emerging outbreaks, in order to prevent further negative impact, the aim of this study is to evaluate the effects of COVID-19 home confinement on the food habits, lifestyle and emotional balance of the Spanish population.

## 2. Materials and Methods 

### 2.1. Participants and Study Design

In Spain, COVID-19 lockdown became obligatory on 14 March. An online cross-sectional study was conducted in the month before lockdown finished, (from 28 May to 21 June), in order to obtain representative results for the entire period of confinement. The target population was Spanish people over 18 years old, living in Spain or abroad during the confinement. We also classified participants depending on the number of inhabitants in the home city (<5.000, 5.000–20.000, 20.000–100.000, 100.000–300.000, >300.000). Digital informed consent was obtained from participants who confirmed they were over 18 years old and were willing to participate voluntarily prior to filling in the online survey. The study was conducted in full accordance with the principles of the Declaration of Helsinki. The study protocol was approved by the Ethics Committee of Francisco de Vitoria University (15/2020).

### 2.2. Data Collection

**Figure 1:**
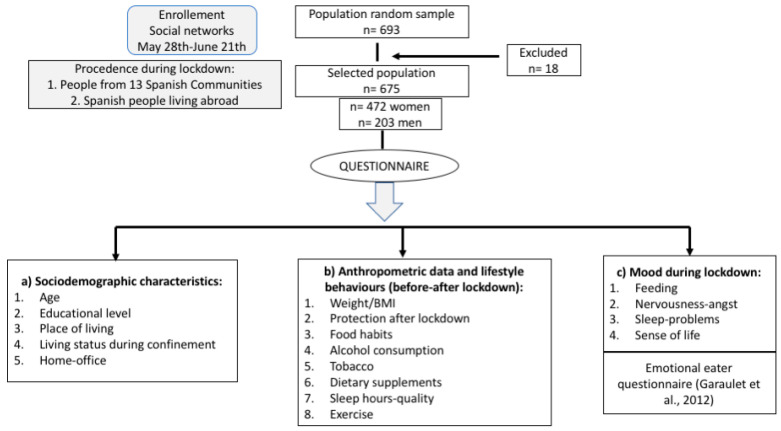
Flow chart of the study participants and blocks of the electronic questionnaire.

Study participants were recruited using social networks such as email, WhatsApp, Twitter, Facebook and Instagram. The “snowball” sampling method was used to recruit more participants. The online survey was made available via social media, and randomly dispersed to as many people as possible during the last month of lockdown. The data were collected via electronic anonymous questionnaire using Google Forms (Google LLC, Menlo Park, CA, USA), consisting of 59 questions about dietary habits and lifestyle, to compare these during and before the lockdown (Figure 1). 

The electronic questionnaire (Appendix A) was divided into different blocks to assess (Figure 1):

- Socio-demographic characteristics: age, gender, educational level, place of living during confinement, living status during confinement (alone, 1 person, 2 people, 3 people, >4 or more people) and home office (yes or no).
- Lifestyle behaviors adopted during lockdown with respect to pre-epidemic conditions:
  1. Anthropometric data. Body Mass Index (BMI) calculated as body weight in kg divided by height in meters squared; height and weight before and during confinement. According to the criteria of the World Health Organization, the BMI was categorized as: underweight (below 18.5 kg/m^2^), normal (18.5–24.9 kg/m^2^), overweight (25.0–29.9 kg/m^2^) and obese (above 30.0 kg/m^2^) [18].
  2. Protection used during lockdown (no use of protection, handwashing, gloves, mask, protective screen).
  3. Food habits: Current diet; whether quarantine resulted in change in dietary habits, increased food consumption, specific food consumed, snacking, and specific type of cooking during lockdown; daily number of consumed meals and frequency of meals before and during lockdown; sources of food during isolation: methods for purchasing or obtaining food; three foods most often consumed; soft drinks and type consumed.
  4. Frequency and type of alcohol consumption.
  5. Smoking frequency during confinement.
  6. Dietary supplements consumption during confinement
  7. Sleep hours before and during lockdown and sleep quality during confinement.
  8. Exercise before and during the lockdown with respect to the time and intensity dedicated.
- Mood changes during lockdown with respect to feeding, nervousness, sleep-problems, overall feeling about life.

Emotional eater questionnaire (EEQ): a ten-item questionnaire developed to assess what extent emotions affect eating behaviour [19]. The questions had four possible replies: (1) never, (2) sometimes; (3) generally and (4) always. The total score ranged from 0 to 30. The subjects were classified into 4 groups: non-emotional eater (score 0–5), low emotional eater (score 6–10), emotional eater (score 11–20) and very emotional eater (score 21–30).

### 2.3. Data Analysis

Data were checked in Excel file for duplicates and any errors before importing and analyzing using SPSS 22 (IBM, Chicago, IL, USA). Firstly, a descriptive analysis of the socio-demographic characteristics of the respondents was conducted. Data are represented as means ± standard deviation and percentages in parentheses (%) for categorical variables. Shapiro-Wilk test was performed to evaluate variables distribution. Mann-Whitney U and Kruskal-Wallis tests were conducted to compare continuous variables among two or more groups, respectively. Chi square test was calculated to evaluate the association between categorical variables. The estimate of the effect size measure was computed as η^2^ for continuous variables and as Cramer’s V Coefficient (V) for categorical variables. The Spearman correlation coefficient was employed to assess the correlation between variables. The correlation coefficients were interpreted using the following thresholds: trivial (r < 0.1), small (0.1 < r < 0.3), moderate (0.3 < r < 0.5), large (0.5 < r < 0.7), very large (0.7 < r < 0.9) and extremely perfect (0 ≥ 0.9). Results were significant for *p* value <0.05.

## 3. Results

### 3.1. Socio-Demographic Characteristics 

The online survey was collected during the last month of lockdown in Spain. In this cross-sectional study, a total of 693 participants were recruited by social media. 18 participants were excluded due to missing data. Finally, 675 participants with an age range between 18 to 85 years old, were included in this study: 472 men and 203 women. 

The territorial coverage spread to: Andalucía, Canarias, Galicia, Comunidad de Madrid, Castilla la Mancha, Asturias, La Rioja, Comunidad Valenciana, País Vasco, Extremadura, Cataluña, Aragón, Castilla y León. Among the results obtained, 41.6% spent the period of confinement in a large city, 48.7% had pursued university studies and 69.9% of participants have been working form a home office during confinement. Table 1 shows the main general characteristics of the study population.

### 3.2. Anthropometric Data 

In the present study, 31.8% of the respondents were overweight/obese before the lockdown while during this period the figures reached 33.2%, with differences by age (Mann Whitney test, Z = −14.7, *p* < 0.05, η^2^ = 0.17) and sex (Chi Square test, *V* = 0.609, *p* < 0.05) (Table 2). Specifically, 49.7% of the men were overweight/obese during confinement while in the case of the women the figure reached 27.3% (Chi Square test, χ^2^ = 36.8, *p* < 0.05, *V* = 0.618). Furthermore, 38.8% of participants gained weight by an average of 2.57 kg during lockdown, while 31.1% reported lost body weight during confinement, 2.81 kg on average. 

**Figure 2:**
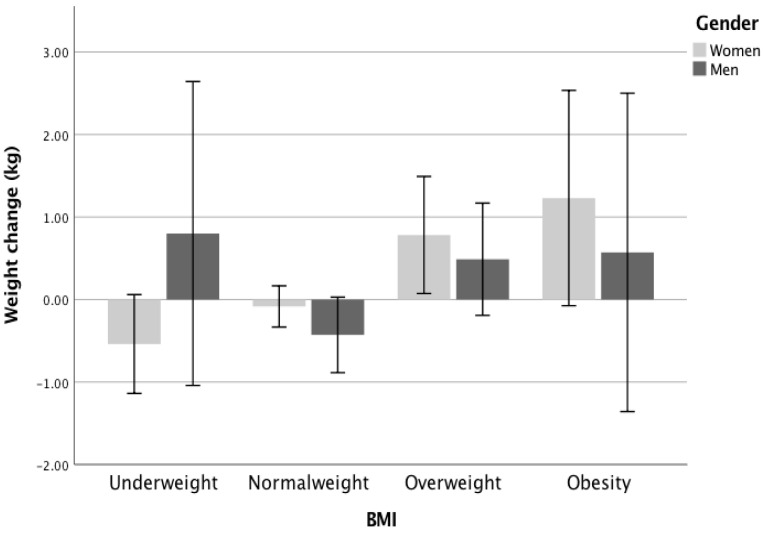
Weight change in different BMI groups and by gender. BMI, Body mass index.

BMI during confinement was positively correlated with age (Spearman’s Rs = 0.32, *p* < 0.01) and this association also remained significant after adjustment for sex, education exercise and sleep. Participants who were obese or overweight gained an average of 1 kg and 0.7 kg, respectively (Kruskal-Wallis test, χ^2^(df) = 21.3(2), *p* < 0.05, η^2^ = 0.296) (Figure 2).

**Figure 3:**
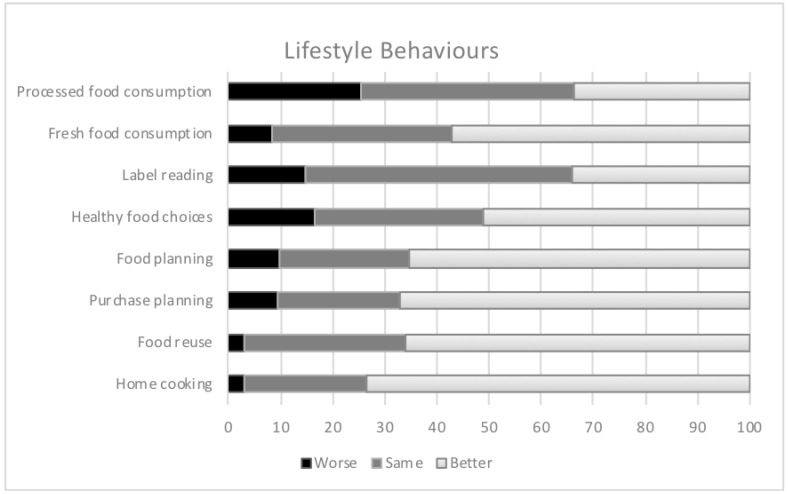
Changes in lifestyle behaviour during confinement. “Better” indicates an improvement in lifestyle behaviors; “same” indicates that lifestyle habits have been maintained; “worse” indicates a worsening of lifestyle behaviors.

Concerning dietary pattern, Figure 3 shows the changes in dietary habits during confinement. In general, 54.4% of participants reported having changed their diet during confinement: 112 (16.2%) declared a change for the worse and 266 (38.4%) claimed to have improved their diet, respectively. Furthermore, an improvement in the perception of eating habits was greater among subjects with greater weight loss during confinement (−0.6 kg vs. +2.1 change for the worse; Kruskal-Wallis test, χ^2^(df) = 51.9(2), *p* < 0.05, η^2^ = 0.121). In the case of perception of food consumption, 19.6% reported having increased food intake while 33.3% reported having decreased it, with differences according to weight variation (Kruskal-Wallis test, χ^2^(df) = 157.6(2), *p* < 0.05, η^2^ = 0.259).

Greater and more efficient preparation of food was declared by 64.2% of participants. In the same way, a positive association was observed between variation in weight and planning of meals and reading labelling (Kruskal-Wallis test, χ^2^(df) = 18.9(2), *p* < 0.05, η^2^ = 0.040). The purchase of snacks and processed foods increased by 39% and 25%, respectively. In addition, the consumption of snacks was associated with weight loss (Kruskal-Wallis test, χ^2^(df) = 22.2(2), *p* < 0.05, η^2^ = 0.036) and BMI (Kruskal-Wallis test, χ^2^(df) = 2.50(2), *p* < 0.05, η^2^ = 0.008). In the case of fresh food, 55.7% of participants stated that they had increased consumption, this being higher among those who lost greater body weight (Kruskal-Wallis test, χ^2^(df) = 7.14(2), *p* < 0.05, η^2^ = 0.012) (Figure 3).

The pattern of number of meals was modified since, during confinement, 23% of respondents declared eating 5 meals per day, whereas previously, this was only reported by 1%. The number of meals was associated positively with BMI during confinement (Kruskal-Wallis test, χ^2^(df) = 9.95(3), *p* < 0.05, η^2^ = 0.287) and with weight gained (Spearman’s Rs = −0.143, *p* < 0.05). These associations remained significant for BMI but not for weight gained after adjustment for sex, age, education, exercise and sleep.

### 3.3. Intake of Dietary Supplements

Regarding the intake of supplements, 20.3% reported consuming them, with a higher incidence among women (22.5% women vs. 15.3% men) (Chi Square test; χ^2^ = 4.5, *p* < 0.05, V = 0.082). In particular, the dietary supplements most consumed amongst the population were vitamin C (7.1%) and vitamin D (4.9%). The users who reported having ingested supplements gained on average 0.62 ± 2.5 kg vs. 0.04 ± 2.7 kg for those who did not take supplements (Mann-Whitney U, Z = −2.47, *p* = 0.014, η^2^ = 0.09). Similarly, 60.6% of the respondents who took supplements reported that they had changed their eating habits in confinement (Chi Square test; χ^2^ = 6.4, *p* < 0.05, V = 0.099).

### 3.4. Changes in Alcohol and Tobacco Consumption 

In the case of alcoholic beverages, only 26.7% of participants reported not ingesting any during the period of confinement and 18.3% consumed them more frequently than usual. Among different alcoholic beverages, beer (47.9%) and wine (20.6%) were the beverages most consumed among drinkers. In the same way, a positive association was observed between variations in weight and alcohol consumption (Kruskal-Wallis test, χ^2^(df) = 12.3(2), *p* < 0.05, η^2^ = 0.035).

Regarding the consumption of tobacco, 17.2% of participants reported smoking and 7.5% smoked more frequently than before confinement. Furthermore, an association between alcohol consumption and the perception of apathy, smoking and anxiety related to food cravings between meals could be observed (Chi Square test, χ^2^ = 0.001, *p* < 0.05, V = 0.132). 

### 3.5. Physical Exercise and Sleep

With regard to sleep, the average number of hours of sleep was 7.02 ± 0.93 h per day, slightly lower than the 7.33 ± 1.22 h per day prior to confinement. Sleep hours during confinement were inversely correlated with BMI (Spearman’s Rs = −0.202, *p* < 0.05) and with age (Spearman’s Rs = −0.209, *p* < 0.05). These associations did not remain significant after adjustment for sex. Furthermore, 39.7% of participants stated that they had poorer quality sleep, in contrast to 19.7% who declared better quality sleep. Regarding difficulty in falling asleep, 28.9% agreed “very much” or “quite agreed”.

Concerning physical exercise, 44.7% of the participants had not performed physical exercise during confinement, with differences by sex (44.7% women vs. 48.7% men) (Chi Square test; χ^2^ = 18.2, *p* < 0.05, V = 164), by age (Kruskal-Wallis test,χ^2^(df) = 28.4(5), *p* < 0.05, η^2^ = 0.098), by BMI (Chi Square test; χ^2^ = 36.7, *p* < 0.05, V = 202), by sleep quality (Chi Square test; χ^2^ = 20.32, *p* < 0.05, V = 0.137) and by snacking between meals (Chi Square test; χ^2^ = 38.4, *p* < 0.05, V = 0.169). Similarly, 20.9% continued the same training pattern and 13.8% began to exercise during this period, with differences by sex (15.5% women vs. 9.9% men) (Chi Square test; χ^2^ = 18.2, *p* < 0.05). Interestingly, differences in weight variation were found depending on the degree of physical exercise during confinement, as those who maintained physical exercise or started exercising in this period lost an average of 0.9 kg and 0.8 kg, respectively, while respondents who did not exercise during lockdown gained on average 1 kg (Kruskal-Wallis test, χ^2^(df) = 69.5(5), *p* < 0.05, η^2^ = 0.099).

Furthermore, of the participants who increased smoking during confinement, 36.5% did not do sports before or after. However, among those who reported smoking less during confinement, 27.8% began exercising during confinement (Chi Square test; χ^2^ = 32.2, *p* < 0.05, V = 126). 

### 3.6. Influences on Emotional Status

During the period of confinement 43% of women and 23.4% of men reported nervousness and distress to a greater extent (Chi Square test, χ^2^ = 38.4, *p* < 0.05, V = 0.239). The perception of boredom and apathy was reported by 33.2% of the participants (33.9% women vs. 31.6% men) (Chi Square test; χ^2^ = 3.1, *p* > 0.05, V = 0.068) and 27.7% declared that they were looking for meaning in their life (30.9% women vs. 20.2% men) (Chi Square test; χ^2^ = 20.5, *p* < 0.05). In addition, 35.6% stated that their mood affected them negatively regarding eating, with differences by gender (38.6% women vs. 28.6% men) (Chi Square test; χ^2^ = 13.7, *p* < 0.05, V = 0.175). The negative impact of mood was greater among participants with obesity during confinement, reaching 62.1%. (Chi Square test; χ^2^ = 22.9, *p* < 0.05, V = 0.130).

Based on the results of the emotional-eater questionnaire, previously validated by Garaulet et al. (2012), most of the participants (40.5% women vs. 37.9% men) were categorized as low emotional eaters. Similarly, 21.8% (22% women vs. 21.2% men) and 11% (13.1% women vs. 5.9% men) were classified as emotional eaters and very emotional eaters, respectively.

The EEQ score was positively correlated with BMI (Spearman’s Rs = 0.24; *p* < 0.05), number of meals (Spearman’s Rs = 0.18; *p* < 0.05) and weight gain during confinement (Spearman’s Rs = 0.19; *p* < 0.05) (Table 3). These associations remained significant after adjustment for sex, age, education, exercise and sleep. Subjects who reported having difficulty “always” or “generally” in stopping eating sweets had a weight gain of 1.7 kg compared to the 0.2 kg lost by those who “never” had difficulty in stopping eating sweet foods during confinement (Kruskal-Wallis test, χ^2^(df) = 10.5(3), *p* < 0.05, η^2^ = 0.021). Likewise, participants who reported “always” eating when stressed, angry or bored gained 1.6 kg during confinement, while those who reported “never” lost 0.3 kg (Kruskal-Wallis test, χ^2^(df) = 26.1(3), *p* < 0.05, η^2^ = 0.036). Surprisingly, weight gain was higher (4.5 kg) in participants who answered “always” and were following a diet during confinement (Kruskal-Wallis test, χ^2^(df) = 16.3(3), *p* < 0.05, η^2^ = 0.0.25). Table 4 shows the variation in body weight classified into several categories.

## 4. Discussion

The main goal of this study was to evaluate the effects of COVID-19 home confinement on the food habits, lifestyle and emotional balance of the Spanish population, in order to try to prevent negative impact on habits and lifestyle in future re-emerging outbreaks. Generally, a quarantine period is associated with stress/depression leading to unhealthy diet and reduced physical activity [20]. Mass media seems to have a significant influence both on knowledge and attitudes; nevertheless, the role of the mass media in health communication is often debated [21]. 

The global outbreak of COVID-19 resulted in restrictive isolation measures in many parts of the world, which led to lifestyle changes during this period of confinement. We reported that 38.8% of respondents increased their body weight by an average of 2.6 kg. These data are similar to those reported in the Spanish population by Sánchez-Sanchez et al., where 37.3% gained between 1 and 3 kg [22]. This result concerning change in body weight is lower compared to the 48.3% observed in a study of the Italian population. However, in the survey research was based on perceived weight gain and not on weight before and during confinement [23]. On the other hand, Zachary et al. reported better results concerning changes in body weight, where 22% reported an increase in this parameter, although this survey only analyzed the period of initial confinement [13]. In this sense, it is important to consider that obese subjects with COVID-19 have a worse prognosis, since they have a higher risk of intensive care unit (ICU) admission and invasive mechanical ventilation [24,25].

An interesting finding of this work was that, on average, hardly any important changes in body weight were observed. However, a great heterogeneity was seen in the variation among the subjects surveyed. Similarly, Sidor et al. found that 29.6% and 18.6% of Polish respondents reported an increase or decrease in body weight, respectively [26]. This lower variability with respect to the present study may be due to the fact that in Poland the stay-at-home order encompassed six weeks, while in Spain it lasted for 14 weeks. It was also found that increased BMI and age correlated with increased weight gain during confinement, as in previous studies [26]. Until now, there has been limited evidence comparing the physiopathology of Covid-19 and obesity, but age is one of the risk factors for hospitalization, therefore an increase in weight in this population group means greater vulnerability to this disease [27,28] Overweight and obesity are continuously increasing in our country, as in the rest in the world. Actually, as reported by Petrova et al., obesity could also be a risk factor not only in older people but in the young, and this issue should be more deeply investigated [29]. 

In our study, 54.4% changed eating habits during lockdown: 25.6% had a worse intake of fast food and, on the contrary, 57.2% had a better intake of fresh food. These results are in line with those reported by Rodríguez-Pérez et al., showing better food choices than before confinement in the north of Spain [8]. Di Renzo et al. found that 53.9% of the Italian population surveyed claimed to have modified their dietary habits, and an increase in unhealthy foods and snacks during home confinement has been observed [23]. Better cooking at home has reported by 73.5% of respondents because of the fact that people spend more time at home. According to the WHO recommendations, this fact can be related to spending more time cooking [3]. In the same way, Ruiz-Roso et al. reported that during confinement families had more time to cook, but apparently did not increase their diet quality [30]. 

Zhao et al. reported that further investigations are needed to measure the consumption of processed foods and its effects on health during future disease outbreaks [31]. It is important to consider the impact of this pandemic situation on lifestyle habits and on the susceptibility to COVID-19 and recovery [32]. We observed that an increase in weight correlated with the number of meals, and this was also reported for other countries [8,13].

Regarding supplement consumption, several dietary behaviors as a response to COVID-19 were identified including an increase in the consumption of vitamins C and D, probiotics and other dietary supplements, similarly to the results observed by Zhao et al. [31]. There is limited evidence for the clinical utility of different supplements and vitamins against COVID-19 [33]. Grant et al. pointed out that vitamin D deficiency could participate in the relationship between obesity and higher susceptibility to infections/death due to Covid-19 [34]. Strikingly in this work, respondents who reported taking supplements were more likely to have greater weight gain during confinement, suggesting that the use of these supplements may be related to a belief in compensating for poor nutrition.

A significant association between alcohol consumption and the perception of apathy and anxiety related to food cravings between meals could be observed. We found that adverse behavior, such as an increase in alcohol consumption compared to before the Covid-19 pandemic (18.8%), was probably due to the stressful situation of lockdown. Similar results were observed by Chodkiewicz et al. (2020), in Poland that during lockdown 28% of individuals reported higher alcohol consumption [35]. In Chile Reyes-Olavarría et al. [36] detected an increase of 30% and Scarmozzino and Visioli reported that alcohol consumption increased by 10.1% in the Italian population [37]. On the contrary, Rodriguez-Pérez et al. [8] and López-Bueno et al. [38] observed a decrease in alcohol consumption in a Spanish population.

Stress is a prominent risk factor in the onset and maintenance of alcohol misuse [39]. The type of beverages consumed more were wine and beer (68.5%) and, less frequently, distilled alcohol. Similar results were obtained in a Spanish population [40]. With regard to our data, nearly one third of participants drank less frequently than before lockdown. As Ozamiz-Etxebarria et al. reported, confinement might tend to produce or exacerbate psychological problems, especially due to alarming information from the news and social media [41]. For this reason, it is important to ensure effective health information.

Regarding tobacco, participants seemed not to have modified their smoking habits, because only 7.7% smoked more frequently than before the confinement when regarding the total smoker population (17.2% of participants were smokers). These results are consistent with previous work that reported smoking habits seeing little difference compared to the period prior to confinement [23,42].

The average sleep duration reported prior to the onset of COVID-19 was 7.1 h/day, as in our results (7.3 h/night), with 50.7% reporting no change in sleep quality. Considering sleeping habits, participants reported maintaining the number of hours of sleep, but with poor sleep quality, which agrees with a previous study in China [43]. These findings are in line with those reported by other previous studies which found that, although sleep hours hardly changed, 40.7% declared a negative change in sleep quality [42]. Furthermore, Cellini et al. also observed a decrease in sleep quality in 11.9%, and a change in sleep–wake rhythms [44]. This effect might be due to the psychological impact of COVID-19, since 18.7% and 21.6% of the Spanish population have been potentially diagnosed with depression and anxiety, respectively [45]. In addition, the isolation situation causes less time spent outdoors, and exposure to sunlight is a determining factor in the regulation of internal circadian rhythms, fundamental to the sleep pattern [46]. Likewise, sleep disruption was associated with a higher BMI. In this sense, poor sleep quality seems to affect the regulation and activity of hormones related to appetite and energy expenditure such as leptin and ghrelin by affecting the function of the hypothalamic-pituitary-adrenal [47].

Due to the Covid-19 pandemic, Pappa et al. in a systematic review suggested a considerable proportion of mood (23.2% depression across ten studies) and sleep disturbances (insomnia 38.9% across five studies) during this outbreak [48]. This author reported the need to establish ways to mitigate mental health risks and adjust interventions under actual and future pandemic conditions. The consequences of sleep deprivation are a misalignment of circadian rhythms leading to increased cortisol levels, associated with an impaired glucose homeostasis, insulin resistance and visceral fat [17].

In times of crisis, the benefits of empowering people to actively preserve their own health should be underlined [49]. After the Spanish Government decreed the state of emergency, it was impossible to practice physical activity outside, and gyms and sport clubs were closed. Exercise has clear health benefits for healthy individuals and during lockdown the maintenance of routine exercising in a safe home environment is important [50]. Our findings showed that 45.9% did not practice physical exercise during lockdown and, in this group, 28.7% did practice physical activity before confinement. Due to this diminished physical activity in our study, an increase of body weight was observed, on average of 1 kg, depending on the degree of physical exercise during confinement. This increase was lower than that observed by Zachary et al., who reported an increase of 2.2 to 4.4 kg, due to less physical activity. Reyes-Olavarría et al. reported in a Chilean population an inverse association with body weight increase [36]. 

In addition to the effect of exercise on body weight as seen in the present survey, it also plays an important role in mental health, because prolonged home confinement can lead a sedentary lifestyle which contributes to anxiety/depression [50,51,52]. Previous studies reported that those subjects in whom confinement negatively affected physical exercise were at greater risk of depression, anxiety and stress [42], which could partly explain the relationship with poor sleep quality among sedentary participants seen in this work.

This study compared mood levels of participants during lockdown with those before the COVID–19 crisis began. It has been observed that the COVID–19 crisis is indeed impacting negatively on the mental well–being of individuals, and the mood of a sample of individuals at quarantine onset was generally poorer compared to the mood before lockdown [53]. Casagrande et al. observed that the COVID–19 crisis is indeed impacting negatively on the mental well–being of individuals [54]. We found that 37.1% of the participants reported suffering from nervousness and stress to a greater extent. Wang et al. and Cao et al. observed an increase of depression, anxiety, and stress in the Chinese population during lockdown [49,55]. 

In the exceptional situation, it is difficult to accurately estimate the emotional consequences of lockdown. There is a bidirectional relationship between foods and moods. The COVID-19 pandemic has caused significant disruption in everyday lifestyle and work routine. Physically and psychologically unhealthy habits have critical implications for quality of life. It has been observed that a stressful situation can develop via overeating and undereating behaviors and, due to this stress situation, people increase their eating of “comfort foods” [56]. We observed that 21.8% and 11% of participants were classified as emotional eaters and very emotional eaters, respectively. Individuals with emotional eating behavior can eat for reasons other than hunger, increasing the intake of palatable foods [57,58].

The EEQ score was directly correlated with BMI, number of meals and weight gain during confinement. Subjects “always” or “generally” having difficulty in stopping eating sweet foods experienced a weight gain of 1 kg and 0.7 kg respectively during lockdown. Antunes et al. suggest the importance of working towards creating strategies to promote healthy eating habits, i.e., not eating more or more often and carefully choosing what to eat [59]. 

Likewise, participants who reported “always” eating when stressed, angry or bored gained 1.6 kg during confinement, while those who “never” did lost 0.3 kg. These results are in line with studies that showed that in situations of greater stress and anxiety people tend to regulate their emotions through food [13,60,61]. Scarmozzino et al. observed that 42.7% of respondents reported that their body weight varied during lockdown due to increase in stress, anxiety and/or boredom [37]. The data related to individuals who always diet is interesting. We observed that this population increased by an average of 4.5 kg, because they reported that their eating had become out of control during lockdown. 

This study has some limitations because sociodemographic factors, lifestyle habits, food intake, drug habits and emotional variables were not studied in depth to avoid an excessively lengthy questionnaire, but these would have obtained a sample more representative of Spanish population. Another limitation was the heterogeneity of the sample in terms of gender. This was the main reason why we used different social networks. However, the number of subjects enrolled in the study was not as large as in studies from other countries, perhaps due to the particularly difficult situation in Spain from March to June 2020. The main limitation of this study was the use of self-declared questionnaires, which can lead to misreporting of data.

Spain is divided into 17 Autonomous Communities and we obtained results from 13, so the sample showed a good range of participation. The Spanish Communities most widely represented were Comunidad de Madrid and Andalucía. The main strength of the present study was that the entire period of lockdown was studied.

## 5. Conclusions

As an overview of our results, some unfavorable nutritional behaviors, decreased physical activity levels, increased sedentary time and weight gain were observed during the three months of confinement in Spain. Lockdown also resulted in a change of habits and, in some cases, eating to compensate for boredom or anxiety with an increase in weight. For some individuals, an increase in alcohol intake was also reported.

On the contrary, we also highlighted individuals with more favorable nutritional behaviors such as an increase in cooked home meals and an increase and/or maintenance in physical activity and eventually loss of weight. Based on these results, suggestions to preserve physical and mental health in future lockdown situations could include recommending individuals to maintain healthy habits, and motivating people to practice or maintain their physical exercise, together with mental distractions such as cooking, dancing, making crafts, gardening, etc., in order to be active and adapting activity to the situation.

However, the present study should be considered as a preliminary description of the data during the Covid-19 lockdown. It is unknown to what degree our data could be generalized to other populations. Our intention is to highlight the need to emphasize the importance of adopting a healthy lifestyle, as the COVID-19 pandemic is ongoing. In the same way, future studies should assess whether these changes have been maintained after the period of confinement.

## Figures and Tables

**Table 1:** General characteristics and demographics of the participants enrolled in the study.

	All (*n* = 675)	Women (*N* = 472)	Men (*N* = 203)	*p*-Value *
Age (years)	39.1 (SD 12.9)	39 (SD 12.8)	39.2 (SD 13.2)	0.888
Education, *N* (%)				0.138
Until middle School	6 (0.9)	2 (0.4)	4 (2.0)	
High School	31 (4.6)	19 (4.0)	12 (5.9)	
Vocational Training	58 (8.6)	36 (7.6)	22 (10.8)	
Undergraduate	329 (48.7)	238 (50.4)	91 (44.8)	
Postgraduate	181 (26.8)	127 (26.9)	54 (26.6)	
PhD	65 (9.6)	45 (9.5)	20 (9.9)	
Others	5 (0.7)	5 (1.1)	0 (0.0)	
Living status during confinement				0.659
Alone	69 (10.2)	50 (10.6)	19 (9.4)	
1 person	161 (23.9)	109 (23.1)	52 (25.6)	
2 people	158 (23.4)	106 (22.5)	52 (25.6)	
3 people	151 (22.4)	106 (22.5)	45 (22.2)	
≥4 people	136 (20.1)	101 (21.4)	35 (17.2)	
Home-office, *N* (%)				0.707
Yes	472 (69.9)	328 (69.5)	59 (29.1)	
No	203 (30.1)	144 (30.5)	144 (70.9)	
Protection used after lockdown *N* (%)				
Mask	667 (99.4)	469 (99.4)	202 (99.5)	**0.044**
Gloves	167 (24.7)	120 (25.4)	47 (23.2)	0.531

* *p*-value by Chi Square test for categorical variables, Mann-Whitney test and Kruskal-Wallis test for continuous variables among two or more groups, respectively. Bold indicates statistical significance (*p* < 0.05).

**Table 2:** Anthropometric parameters: Body Mass Index (BMI) and reported changes in body weight.

	All (*n* = 675)	Women (*N* = 472)	Men (*N* = 203)	*p*-Value *	Effect Size
Weight before (kg)	68.7 (SD 15.3)	63.5 (SD 13.4)	80.5 (SD 13.4)	**<0.001**	0.056
Weight after (kg)	68.8 (SD 15.8)	63.7 (SD 14.2)	80.5 (SD 13.5)	**<0.001**	0.234
Height (m)	170.3 (SD 35.5)	166.7 (SD 41.5)	178.8 (SD 9.9)	**<0.001**	0.024
BMI before (kg/m^2^)	23.9 (SD 4.9)	23.4 (SD 5.2)	25.2 (SD 4.0)	**<0.001**	0.031
BMI after (kg/m^2^)	24.2 (SD 10.8)	23.8 (SD 12.6)	25.2 (SD 4.0)	**<0.001**	0.037
Change in body weight (kg)	0.12 (SD 2.7)	0.15 (SD 2.6)	0.07 (SD 2.8)	0.758	0.001

* *p*-value by Mann-Whitney test. Effect size was computed as η^2^. Bold indicates statistical significance (*p* < 0.05).

**Table 3:** Correlation analysis of anthropometric values with general characteristics and lifestyle.

Variable	Weight Variation		BMI before Confinement		BMI during Confinement	
	Rs	*p*-Value	Rs	*p*-Value	Rs	*p*-Value
Age	0.138	**<0.01**	0.297	**<0.01**	0.318	**<0.01**
Number of meals	−0.055	0.15	−0.089	**0.02**	−0.104	**<0.01**
Sleep (h/day)	−0.064	0.09	−0.204	**<0.01**	−0.202	**<0.01**
EEQ	0.192	**<0.01**	0.185	**<0.01**	0.223	**<0.01**

Data presented as Spearman correlation coefficient. Bold indicates statistical significance (*p* < 0.05). BMI, Body mass index; EEQ, Emotional eater questionnaire.

**Table 4:** Changes in weight during confinement according to different characteristics.

Variable	Number (%)	Weight Change (kg)	*p*-Value	Effect Size
Gender				
Men	472 (69.9)	0.15		
Women	203 (30.1)	0.07	0.758	0.001
Age (years) *			**0.012**	0.089
18–30	167 (24.7)	−0.26		
30–65	487 (72.1)	0.27		
>65	21 (3.1)	−0.14		
Living alone			0.913	0.001
No	69 (10.2)	0.12		
Yes	606 (89.8)	0.13		
BMI before confinement				0.02
≤25	455 (67.4)	0.14		
>25	220 (32.6)	0.09	0.41	
Dietary				0.009
supplements				
Yes	137 (20.3)	0.01		
No	538 (79.7)	0.62	**0.013**	
Active smokers				0.003
Yes	116 (17.2)	0.33		
No	559 (82.8)	0.08	0.985	
Active alcohol drinkers				0.002
Yes	185 (27.4)	0.13		
No	490 (72.6)	0.12	0.758	
Sleep during confinement			**0.04**	0.006
<7 h/day	143 (21.2)	0.47		
≥7 h/day	532 (78.8)	0.03		
Exercise during confinement				0.02
Yes	559 (82.8)	−0.05		
No	116 (17.2)	0.9	**0.01**	
EEQ *				0.031
Non-emotional eater	186 (27.6)	−0.37		
Low emotional eater	268 (39.7)	−0.06		
Emotional eater	147 (21.8)	0.64		
Very emotional eater	74 (11.0)	1.03	**<0.001**	

*p* by Mann-Whitney test, excepting * p by Kruskal-Wallis test. Bold indicates statistical significance (*p* < 0.05). Effect size was computed as η^2^. BMI, body mass index; EEQ, Emotional eater questionnaire.

## Appendix A

SECTION A

**Q1. How old are you?**

**Q2. In which Community or Autonomous City have you spent the period of confinement?**

**Q3. In which municipality have you spent the period of confinement?**

**Q4. Gender:** *Female/Male*

**Q5. What is your level of education?** *Primary school/Secondary school/Vocational training//University/Master’s degree/PhD/Others.*

**Q6. How many people have you lived with at home during your confinement?** *Alone/1 person/2 persons/3 persons/4 or more persons*

SECTION B

**Q7. Weight before the pandemic:**

**Q8. Current body weight (kg):**

**Q9. Height (cm):**

**Q10. Have you been on telework/online classes during your confinement?** *Yes/No*

**Q11. Do you use personal protective equipment when you leave home? Select the ones you use?** *Mask/Gloves/Protective screens*

**Q12. Where did you eat before confinement?** *House/cafeteria/homemade*

**Q13. In general, how do you think the following habits have been affected by the confinement situation?**

**Q13.1 Elaboration of meals at home:** *For better/for worse/equal*

**Q13.2 Reuse of surplus food:** *For better/for worse/equal*

**Q13.3 Shopping list planning:** *For better/for worse/equal*

**Q13.4 Meal planning:** For better/for worse/equal

**Q13.5 Healthy food choices:** *For better/for worse/equal*

**Q13.6 Label reading:** For better/for worse/equal

**Q13.7 Purchase of snacks:** For better/for worse/equal

**Q13.8 Fresh food:** For better/for worse/equal

**Q13.9 Processed/Prepared Foods:** *For better/for worse/equal*

**Q14. What type(s) of culinary preparations have you increased during confinement? (fried food, spoon dishes, sandwiches, grill, steam, prepared food, etc.):**

**Q15. List 3 foods/products that you have deemed indispensable during the confinement:**

**Q16. Have you started taking any vitamin/mineral supplements?** *Yes/No*

**Q17. If yes, which supplement(s)?**

**Q18. Do you smoke more than before confinement?** *I do not smoke/Yes, I smoke more/No, I smoke the same/No, I smoke less*

**Q19.Do you drink alcoholic beverages more often than before confinement?** *I do not drink alcoholic beverages/No, I just drink as often/No, I drink less often/Yes, I drink more often*

**Q20. In case you consume more alcoholic beverages, which one(s)?**

**Q21. Do you drink more soda than before confinement**? *I don’t drink soda/Yes, I drink more soda than before/No, I drink just as often/No, I drink less often*

**Q22. In case you consume more soft drinks, which one(s)?**

**Q23. How many hours of sleep did you get on a typical day before confinement?**

**Q24. How many hours do you usually sleep these days?**

**Q25. In general, how do you rate the quality of your sleep compared to before confinement?***Better*/*Same*/*Worse*

**Q26. Have you been able to keep up with the exercise during confinement?** *I didn’t do sport then and I don’t do it now/Yes, I’ve kept up my training/Yes, but with less intensity/Yes, but for less time/No, I couldn’t keep up with the exercise/I have begun to exercise during confinement*

**Q27.Has the time spent on housework changed during confinement?** *It has decreased/It has remained the same/It has remained the same*

**Q28. Do you consider that you have changed your eating habits during confinement?** *Yes/No*

**Q29. How many intakes do you make per day of these top five? Check the ones you usually do.**

**Q29.1 Before confinement:** *Breakfast/Mid-morning/Lunch/Snack/Dinner/Bedtime snack*

**Q29.2 During confinement:** *Breakfast/Mid-morning/Lunch/Snack/Dinner/Bedtime snack*

**Q30. Do you think you are eating better or worse than before?** *Better/Worse/Same*

**Q31. Do you think you are eating more or less than before?** *More/Less/Same*

**Q32. Does it sting between hours more or less than before confinement?** *More than before/Less than before/Same as before*

SECTION C

**Q33. Do you think there is a relationship between food and health?** *Yes/No*

**Q34. Do you like to eat?** *Yes/No*

**Q35. Do you consider that your state of mind during confinement has influenced your diet?***Yes, in a positive way*/*Yes, in a negative way*/*It has not influenced*

**Q36. Indicate your degree of agreement with the changes in mood you have felt since the confinement began:**

**Q36.1 Nervousness/anxiety:** *Nothing/A little bit/Regular/Quite a lot/Very much in agreement*

**Q36.2 Boredom/Apathy/Irritability:** *Nothing/A little bit/Regular/Quite a lot/Very much in agreement*

**Q36.3 Difficulty in falling asleep:** *Nothing/A little bit/Regular/Quite a lot/Very much in agreement*

**Q36.4 Looking for the meaning of my life:** *Nothing/A little bit/Regular/Quite a lot/Very much in agreement*

Emotional eater questionnaire (EEQ) Garaulet

**Q37. Do you weight scales have a great power over you? Can they change your mood?** *Never/Sometimes/Generally/Always*

**Q38. Do you crave specific foods?** *Never/Sometimes/Generally/Always*

**Q39. It is difficult for you to stop eating sweet things, especially chocolate**. *Never/Sometimes/Generally/Always*

**Q40. Do you have problems controlling the amount of certain types of food you eat?** *Never/Sometimes/Generally/Always*

**Q41. Do you eat when you are stressed, angry o bored?** *Never/Sometimes/Generally/Always*

**Q42. Do you eat more of your favourite food and with less control when you are alone?** *Never/Sometimes/Generally/Always*

**Q43. Do you feel guilty when eat “forbidden” foods, like sweets or snacks?** *Never/Sometimes/Generally/Always*

**Q44. Do you feel less control over your diet when you are tired after work at night?** *Never/Sometimes/Generally/Always*

**Q45. When you overeat while on a diet, do you give up and start eating without control, particularly food that you think is fattening?** *Never/Sometimes/Generally/Always*

**Q46. How often do you feel that food controls you, rather than you controlling food?** *Never/Sometimes/Generally/Always*
